# A modified protocol merging two published techniques for computer guided zygomatic implants surgery: a technical note

**DOI:** 10.1186/s12903-024-05010-1

**Published:** 2024-10-24

**Authors:** Omar Effat Mokhtar Abouzeid, Ramy M. Gaber, Haitham A. Maergy, Hossam El-Dien Hany, Karim M. Abdelmohsen, Marwa A. Elkassaby, Moustafa M. Taha

**Affiliations:** 1 Oral and Maxillofacial Surgery Department, Faculty of Dentistry, Ain Shams University, Cairo, Egypt; 2 Private practice, Cairo, Egypt

**Keywords:** Zygomatic implants, Computer guided surgery, Double sleeve technique, Drilling guides

## Abstract

**Objectives:**

Zygomatic implant surgery can be difficult due to the limited intraoperative visibility of the surgical field and the complex anatomy of the zygomatic bone, which could lead to serious complications. This study aims to assess the accuracy of zygomatic implants placement using computer-guided surgical templates.

**Materials and methods:**

A total of 13 zygomatic implants were placed in four participants. Double-sleeve drill guides were used with the help of computer-guided surgical templates designed with a lateral window. The accuracy was evaluated by measuring the linear deviations regarding the implants’ platforms and apices’ positions in addition to the angular deviations. Moreover, deviations of both implants from three fixed planes of space were measured.

**Results:**

The mean linear deviation at platforms was 2.44 mm ± 1.57 and at the apices 2.32 mm ± 1 while the mean angular deviation was 3.6˚ ± 1.92. Differences at the entry points were 0.43 ± 1.79 mm, 0.39 ± 1.12 mm, and − 0.54 ± 2.00 mm from the mid-sagittal, horizontal, and coronal planes respectively. Differences at the exit points were − 0.75 ± 1.25 mm, -0.06 ± 1.09 mm, and 0.63 ± 1.24 mm from the same planes respectively. Within all planes, there was no statistically significant difference.

**Conclusion:**

Given the limitations of this study, the use of the computer-guided surgical templates augmented by the double sleeve drill guides allowed favorable control over the tip of the long surgical drill away from vital structures during the zygomatic implant osteotomy. It also allowed control over alveolar crest osteotomy and its placement in a favorable prosthetic position. Overall, this protocol should be considered for further research and improvement to allow more predictable surgical outcomes while preventing the occurrence of complications. Before conducting this study, the protocol was reviewed and approved by the Research Ethical Committee of Faculty of Dentistry, Ain Shams University in meeting no. (105), on 15th of July 2020 with the application no.: (FDASU-RecD072029).

## Introduction

Since *Branemark* first introduced zygomatic implants in 1988, many modifications have been reported regarding their designs, surgical approaches and loading protocols. With high survival and success rates documented in the literature, zygomatic implants indications have expanded to include severely resorbed maxillary ridges with insufficient bone for conventional implant placement. The strength of the anchorage in the zygoma compensates for the poor quality of the atrophied maxillary bones. In many cases, grafting is not an option due to the residual morphology of the atrophic maxillae following bone resorption. Given the opportunity for immediate loading after engaging stable cortical bone and abating the need for grafting procedures, the decision to utilize zygomatic implants for edentulous maxillary rehabilitation has been influenced by the shortened treatment time [1–3].

However, zygomatic implant surgery can be difficult due to the limited intraoperative visibility of the surgical field and the complex anatomy of the zygomatic bone. The curvature of the sinus lateral wall and the sinusoidal shape of the zygomatic posterior wall make it challenging to control the long surgical drilling path and the zygomatic fixture placement. This imposes a great challenge during free-hand osteotomy, especially for less experienced surgeons placing quad zygomatic implants leading to potential serious complications such as penetration of the orbital cavity and the infratemporal fossa [2, 4, 5].

In 2000, the use of computer-assisted navigational system was initially introduced to zygomatic implant rehabilitations by *Schramm et al.* and *Watzinger et al.* to decrease the incidence of these complexities [6, 7]. Afterwards, *Vrielinck et al.* were the first to report an in vivo study on the precision of zygomatic implants placement in 2003 to validate the use of personalized static computer guided drilling templates [8]. Compared with the conventional free-hand technique, such implementations have shown a greater degree of accuracy in transferring the planned implant position to the surgical site [4, 5, 9, 10]. Nevertheless, templates used for conventional implants were considered inadequate for this task. Having only a single guide crestal sleeve, this approach does not adequately control the stability and direction of the apical portion of the drill when the site is prepared for the placement of the longer zygomatic implants. Additionally, these long drills are subjected to increased mechanical stresses, which could eventually compromise the stability of the template [2, 11] .

To increase the accuracy of computer-aided zygomatic implant surgeries, different concepts and techniques have been proposed. In 2016, *Chow* introduced a novel drill guide with double sleeves, designed to stabilize the long zygomatic drills [11]. Later, multiple researchers utilized his concept after applying several modifications to minimize the deviations as much as possible [2, 12]. Still, none of these studies has provided the evidence that is strong enough to be considered as the gold standard with the optimum degree of accuracy [5]. In this study, our aim was to use and evaluate a modified protocol, combining the previously described device and surgical guide design. We used the double-sleeve drill guide proposed by *Chow* with the help of a computer-guided surgical template designed with a lateral window as suggested by *Rinaldi et al.* [11, 13].

## Materials & methods

This prospective trial enrolled patients seeking maxillary arch rehabilitation using dental implant treatment. Sample size calculation was conducted using G*Power 3.1.9.4 Software based on data obtained from a previous study by *Vrielinck et al.* that reported 5.14˚ mean angular deviation and 2.59 standard deviation of zygomatic implants placed by CT-based planning. The effect size was calculated to be 0.82. A sample size of 13 implants was required to ensure that the 95% confidence interval estimate of the mean angular deviation of zygomatic implants placed by virtual planning and surgical guides was within 3˚ of the true mean. The power of t-test was estimated to be 80%, using a two-tailed significance level of 5%.

Before conducting this study, the protocol was reviewed and approved by the Research Ethical Committee of Faculty of Dentistry, Ain Shams University in meeting no. (105), on 15th July 2020 with an application no.: (FDASU-RecD072029). Patients were recruited at the Department of Oral and Maxillofacial Surgery, Faculty of Dentistry, Ain Shams University and at the Egyptian Zygoma Implant Institute (EZII) clinic. The systemic health condition of all the participants was recorded and they were selected according to the following criteria fulfillment:

### Inclusion criteria

1. 18-year-old (or older) patients who can understand and sign an informed consent.
2. Patients in need of maxillary arch rehabilitation using dental implants.
3. Patients with severe alveolar bone atrophy in both Bedrossian zone II & III or all three zones [14].
4. Patients with a history of unsuccessful bone grafting, failure of conventional implants, or refusal to undergo further bone augmentation procedures.
5. Patients with zygomatic bone of a 15 mm minimum width [15, 16] .
6. Patients with good compliance and oral hygiene habits.

### Exclusion criteria

1. Patients with cardiovascular disease, pulmonary disease or medical systemic condition that might hinder the fitness for general anesthesia (ASA III, IV, V and VI).
2. Patients with conditions contraindicating implant placement (e.g.: radiation to the head and neck, intra-venous bisphosphonates, uncontrolled Diabetes mellitus).
3. Acute maxillary sinus infection or untreated maxillary sinus cyst.
4. Heavy Smokers (more than 20 cigarettes per day).
5. Restricted mouth opening (less than 3 cm interincisal distance).

In order to accurately formulate an optimal virtual treatment plan, the pre-operative Digital Imaging and Communications in Medicine (DICOM) files obtained from the multi-slice computer tomography (MSCT) scan were imported into *Blue Sky Plan* software. The acquired scans had the following parameters: (i) axial images only, (ii) no gantry tilt, (iii) slice thickness 0.625 mm, (iv) slice distance 0.625 mm, (v) field of view extending from the Glabella superiorly to the mandibular arch inferiorly, and (vi) bony window. The software was used to manipulate CT images, virtually plan for implant placement, design surgical guides, and export 3D virtual volumes as Standard Tessellation Language (STL) files. The desired implant positions were identified through the software to achieve the best possible functional and prosthetic position possible.

**Fig. 1:**
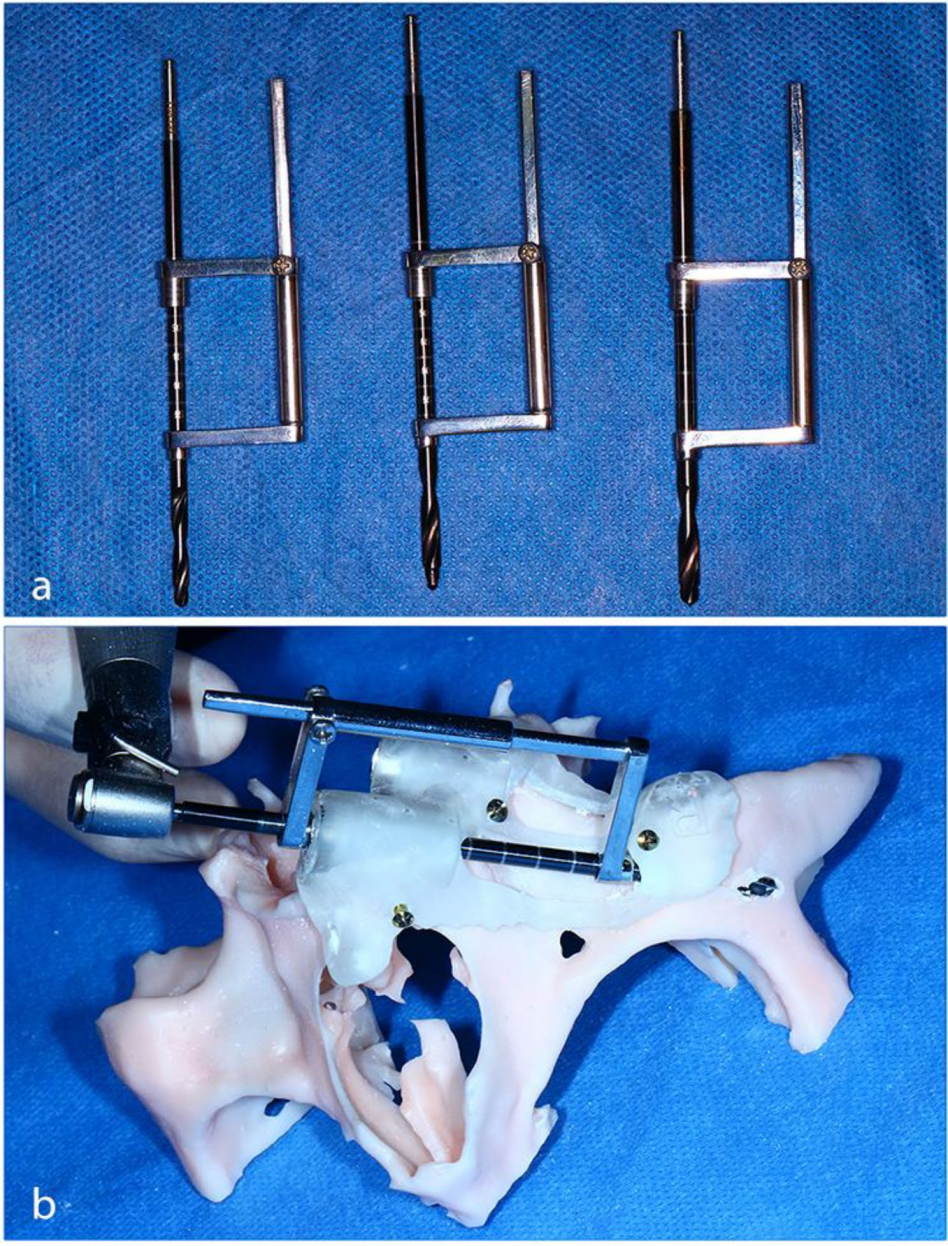
(**a**) Showing the co-axial alignment of The Double-sleeve drill guides. (**b**) Using the final drill for the zygomatic osteotomy of the anterior implant

During the guide-designing process, a cut-off window in the surgical guide was created slightly wider than the zygomatic implant and along its path. In conjunction with the computer-guided surgical templates, a customized double-sleeve drill guide kit – called the ‘zygoma drill guide’ – was used in this study. The zygoma drill guide was designed using CAD software with a few modifications to the original design developed by *Chow.* It was milled and produced out of stainless steel. Disassembly for cleaning and autoclaving was considered. Our zygoma drill guide kit composed of a set of three guides, one for each of the different-diameter drills contained in the manufacturer drilling kit. Figure 1*(a)*.

Each drill guide consisted of two heads in co-axial alignment, which guided the drill at both entry points. These two heads were metal rings each with an internal diameter 0.15 mm larger than its corresponding drill. The two heads were connected by a sliding arm consisting of a rectangular rod enclosed in an open-ended tube. The maxillary entry head was inserted into the metal guiding sleeve fitted in the surgical template. The exit head was positioned at the base of the surgical template fitted in its lateral cut-off implant window resting on the sleeve-like or half-sleeve extension. When inserted into the surgical template, the drill went through the first entry head at the drill sleeve and engaged the second zygomatic head before penetrating the zygomatic bone.

The surgical guides along with the 3D model of the mid-face were exported in STL format. The exported files were then 3D-printed in clear photopolymer resin using the Any-cubic Photon Mono X (MSLA) 3D printer. Rehearsal mock surgeries for all the cases were performed on the 3D model prior to the surgical procedures. During these rehearsals, the same protocol and steps to be performed in the actual surgery were followed strictly. Figure 1*(b)*.

Prior to surgery, the surgical templates were submerged in a basin containing 2.4% activated glutaraldehyde solution for 20–30 min for high-level disinfection, followed by sterile saline rinse.

The surgeries were performed under general anaesthesia with nasotracheal intubation. After complete exposure of all anatomical landmarks, the surgical templates were positioned in place. Once the templates were properly seated, they were secured in place using four to five 2.0 screws ranging from 10 to 14 mm in length.

The zygomatic implants used were JDZygoma implants (JDentalCare, Madona, Italy) which were available with main implant diameter of Ø 4.3 mm, maximum implant diameter of Ø 4.5, an implantable length of 18 mm, a tip diameter of 2.15 mm and lengths ranging from 30 mm to 60 mm in 2.5 mm increments. Osteotomy drilling was performed in a two-step approach. First, the manufacturer’s key-less guided surgical drilling kit was used to complete the alveolar part of the osteotomy. Sequential drills entered the maxilla palatally puncturing the alveolar crest to reach the buccal side. Completion of the alveolar osteotomy was performed using the drill equivalent in diameter to the final drill of the zygomatic implant drilling kit, which is manufactured by the same company.

**Fig. 2:**
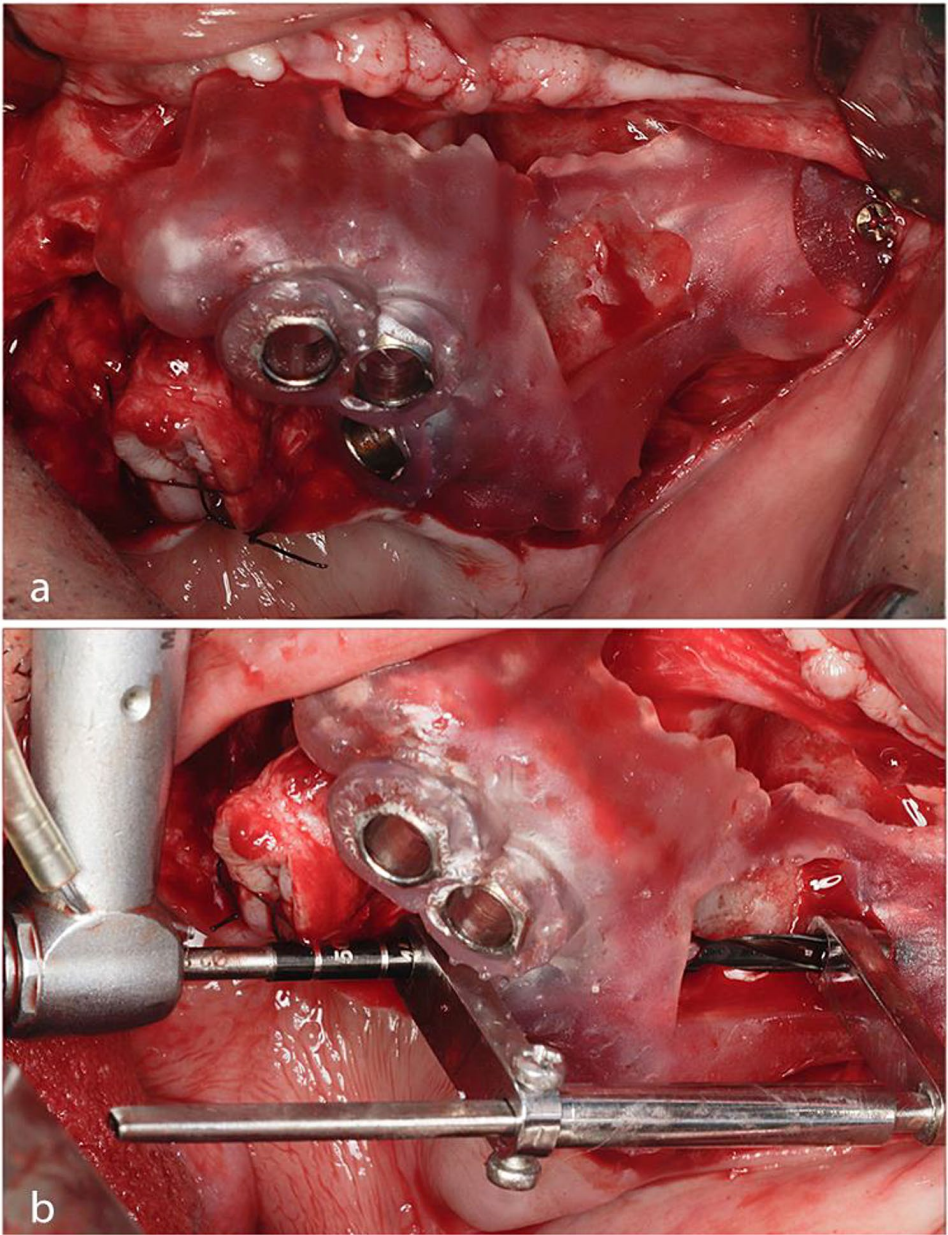
(**a**) Showing the surgical template adaptation and fixation over the exposed bony surfaces from frontal view. (**b**) Using the initial drill for the zygomatic implant osteotomy

During the second part of the osteotomy, the three zygoma drill guides were used after fitting their first entry heads in the metal sleeves of the surgical template. The long drill tips were guided properly to its second entry point in the zygomatic bone. The drill was visualized through the window created in the surgical templates until it reached the zygomatic bone to prepare the implant osteotomy. Figure 2.

**Fig. 3:**
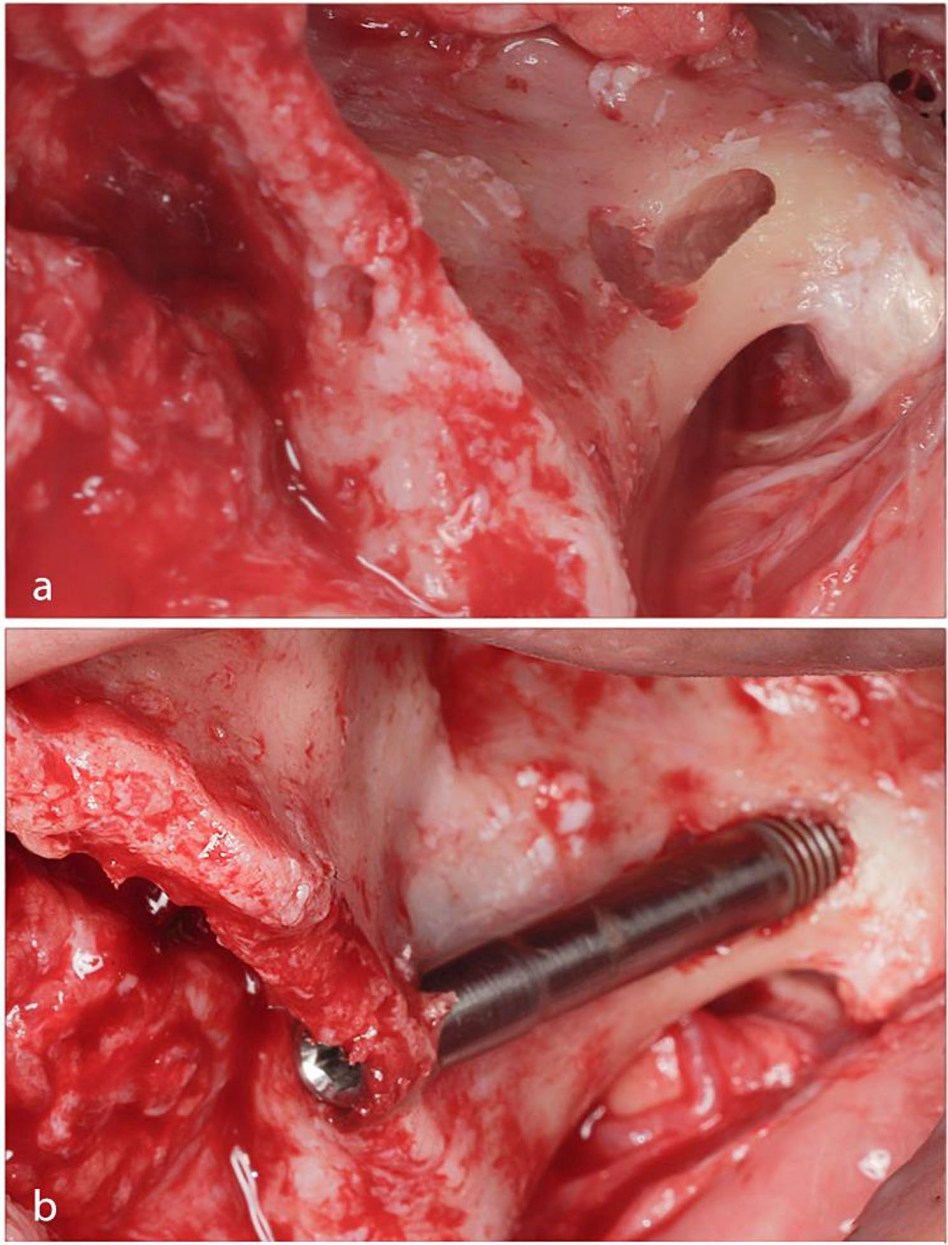
(**a**) Showing the tunnel-shaped osteotomy. (**b**) Free-hand placement of the zygomatic implant after surgical template removal

Upon completing the osteotomies and removing the surgical guide, a depth gauge was used to detect the depth and direction of the osteotomy confirming the zygomatic implant length. Afterwards, each zygomatic implant was manually placed with implant insertion torque not exceeding 80 N/cm until its tip reached the inferior border of the zygomatic osteotomy exit point. Figure 3.

Finally, buccal fat pads were used to cover the extra bony shafts of the implant, enhancing soft tissue quality around, and the flaps were repositioned and sutured with 4 − 0 polyglycolic acid sutures. Within 4–5 days, the patient was called back for a follow-up visit to assess the healing process and to exclude any chances of wound infection. Ten days later, the sutures were removed upon complete wound healing. For the radiographic assessment of postoperative implants positions, a 2-week postoperative facial bones MSCT scan was requested from all patients. The postoperative CT scans were required to fulfil the same criteria as the preoperative CT scans and to be performed at the same radiology center.

After superimposition, fixed landmark points were determined on both the virtually planned implants and the actual postoperative implants in the exact same positions. These points, which were determined as entry points at the implant platforms and exit points at the implant apices, were marked at both ends of each implant. The three planes of space, namely, the Mid-Sagittal Plane (MSP), the Frankfort Horizontal Plane (FHP) and the Coronal Plane (CP), were determined in relation to the skull. Measurements of the distances from the two points of each implant to the three planes of space were calculated for both the preoperative virtually planned implants and the actual postoperative implants. In addition, the direct distance between each entry and exit point on the virtually planned implants and its equivalent point in the actual postoperative implants was measured directly. Furthermore, angular deviation was recorded by measuring the angle between the two lines representing the long axes of the virtually planned implant and the actual implant.

By comparing all the measurements, we determined the degree of accuracy of the applied virtual surgical planning intraoperatively three-dimensionally and detected how accurate the computer-guided surgical templates were in transferring the virtual plan to the operating room, which influenced the final implants positions. Categorical data were presented as frequency and percentage values. Numerical data were presented via the mean with 95% confidence intervals, standard deviations, and minimum and maximum values. The data were explored for normality by checking the data distribution using Shapiro-Wilk test. Data showed parametric distribution and were analyzed using paired t-test. Inter- and intra-observer reliability were analyzed using intra-class correlation coefficient (ICC). The significance level was set at *p* ≤ 0.05 within all tests. Statistical analysis was performed with R statistical analysis software version 4.3.0 for Windows. The datasets used and/or analyzed during the current study are available through the corresponding author upon reasonable request.

## Results

A total of 13 implants were placed for four participants seeking maxillary arch rehabilitation using dental implant treatment. One female patient and three male patients were included in the study. The male patients were aged 37, 58 and 74 years, and the female patient was 56 years old. The age range was 37 to 74 years with a mean age of 56 years old. The implant distribution was four implants for each patient with the exception of one patient who received only one zygomatic implant as part of the research. For this patient, two additional conventional implants were placed ipsilaterally in conjunction with this single zygoma implant, having no conflicts with the research. The patients’ characteristics are shown in Table 1.

**Table 1:** Clinical characteristics of the patients

Clinical Characteristics		*Total (N = 4)*
Age (years)		56.25 ± 15.15
Gender	Male	3 (75%)
	Female	1 (25%)
Previously Failed Implants	Yes	2 (50%)
	No	2 (50%)
Smoking Status	Yes	2 (50%)
	No	2 (50%)
Opposing Arch	Teeth	0 (0%)
	Implants	3 (75%)
	Mixed	1 (25%)

**Fig. 4:**
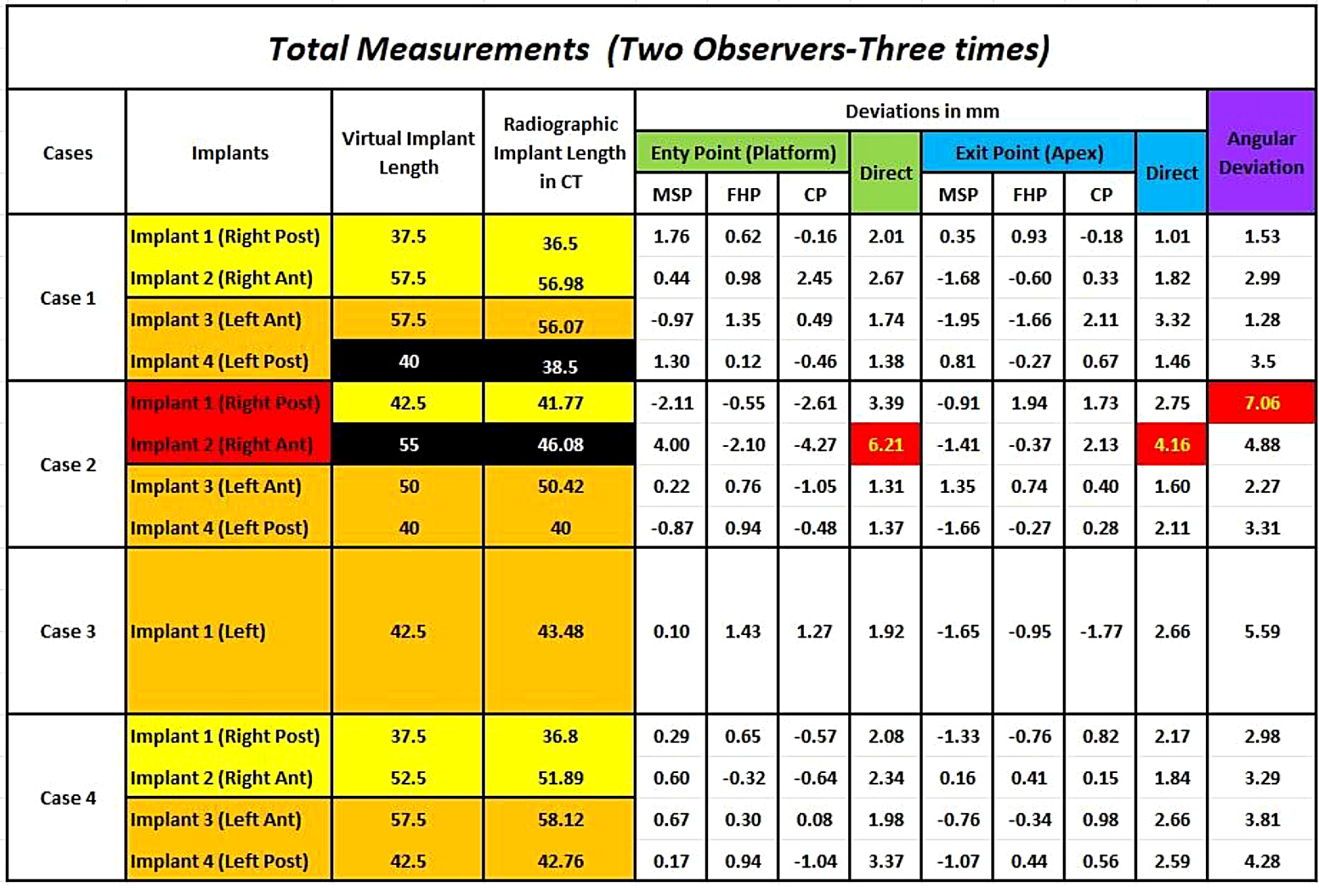
Measurements of the deviations detected for all zygomatic implants included in the study

No significant problems or complications impeded the completion of the surgeries. In one case, the anterior implant sleeve of the right-side surgical guide fractured during drilling. However, this situation did not affect the positioning of the implant during placement as the fracture took place during the final drill osteotomy. Among all the implants, only two implants were placed in different lengths than what was planned in the virtual treatment. One Implant was 37.5 mm instead of 40 mm (Case 1, Implant 4) while the other was 47.5 mm instead of 55 mm (Case 2, Implant 2). Measurements of all deviations are shown in detail in Fig. 4. All the patients had an uncomplicated immediate postoperative period with no major problems. As expected, tension, discomfort, and facial edema of variable degrees were experienced by the patients postoperatively.

Radiographic evaluation at the level of entry points was performed post operatively for all patients and compared to the preoperative plan. The deviation was non-statistically significant (*P* = 0.49) in the MSP with a 0.43 mm measurement difference (SD = ± 1.79). In the FHP, the deviation was 0.39 mm (SD = ± 1.12) with no statistical significance (*P* = 0.32). The difference measured in the CP was − 0.54 mm (SD = ± 2) which was not statistically significant (*P* = 0.44).Table 2.

**Table 2:** Differences between virtual and post operative measurements (entry points)

Plane	(mean ± SD) (mm)		*p*-value	Difference (mean ± SD) (mm)
	Virtual plan	Post-operative		
**MSP**	16.07 ± 5.32	16.50 ± 5.12	**0.491ns**	0.43 ± 1.79
**FHP**	30.48 ± 4.05	30.87 ± 4.62	**0.321ns**	0.39 ± 1.12
**CP**	60.30 ± 8.96	59.77 ± 9.24	**0.444ns**	-0.54 ± 2.00

*; significant (*p* ≤ 0.05) ns; non-significant (*p* > 0.05)

Moreover, differences between virtual and post-operative measurements at the exit point level were also measured. In the MSP, a -0.75 mm (SD = ± 1.25) deviation was detected with no statistical significance (*P* = 0.1). A non-statistically significant (*P* = 0.87) difference of -0.06 (SD = ± 1.09) was measured in the FHP. For the CP, the measurements showed 0.63 mm (SD = ± 1.24) non-statistically significant (*P* = 0.16) difference. Table 3.

**Table 3:** Differences between virtual and post operative measurements (exit points)

Plane	(mean ± SD) (mm)		*p*-value	Difference (mean ± SD) (mm)
	Virtual plan	Post-operative		
**MSP**	49.22 ± 1.92	48.47 ± 1.82	**0.109ns**	-0.75 ± 1.25
**FHP**	3.41 ± 1.88	3.35 ± 2.35	**0.879ns**	-0.06 ± 1.09
**CP**	51.54 ± 5.43	52.17 ± 4.53	**0.164ns**	0.63 ± 1.24

*; significant (*p* ≤ 0.05) ns; non-significant (*p* > 0.05)

By measuring the linear distance between the actual and virtual implants at the level of both entry and exit points, the descriptive statistics for direct linear deviation were calculated. The 95% confidence of interval revealed that deviations at the platforms ranged from 1.42 mm to 3.47 mm and the calculated mean deviation was 2.44 mm ± 1.57 mm. However, the full range of deviations at the entry level was 1.31 mm at minimum and 6.21 mm at maximum. With respect to deviations at the exit level, the minimum measurement was 1.28 mm, and the maximum was 4.16 mm with a mean of 2.32 ± 1 mm. The 95% confidence interval ranged of 1.67 mm to 2.97 mm. Table 4.

After measuring the angle formed between the two lines joining the entry and exit points of both virtual and actual implants, the descriptive statistics for angular deviation were calculated, and the mean angular deviation was 3.6 ± 1.9˚ with a minimal and maximal deviation of 1.28˚, 7.06˚ respectively. The lower margin of the 95% confidence of interval was 2.35˚ and its upper margin was 4.86˚. Table 4.

**Table 4:** Descriptive statistics for direct deviation and angular deviation

Parameter	Mean	95% confidence interval		SD	Min	Max
		Lower	Upper			
**Entry point**	2.44	1.42	3.47	1.57	1.31	6.21
**Exit point**	2.32	1.67	2.97	1.00	1.01	4.16
**Angular deviation**	3.60	2.35	4.86	1.92	1.28	7.06

## Discussion

The precision of any surgically guided procedure depends largely on the ability to accurately position the surgical template on top of the bone. Thus, our goal was to secure the best possible stability when designing the surgical templates by covering most of the maxillary bony surfaces exposed during the surgery [8]. Aiming to achieve a tripodal bony support, we added a third mounting point along with the zygomatic bone and palatine process of the maxilla by extending our templates anteriorly to engage the anterior nasal spine. We believe that this prevented the guide mobility around any of its three axes providing the greatest stability and placement accuracy. To maintain that stable position during the procedure, the guides were fixed into position using four or five osteosynthesis screws [8].

The cut-off window created in the surgical guide was carefully positioned to expose the point where the drill or the implant should exit the maxilla. Additionally, its proper positioning indicated the zygomatic entry point with a small incomplete sleeve-like extension from the surgical guide. This allowed the operator to visualize the implant trajectory and to monitor and evaluate the whole drilling procedure. This protocol was originally reported by *Rinaldi et al.* in 2019 [13].

For each case, a 3D midface model was printed prior to the surgical procedure. As recommended by *Aparicio et al.*, a pre-operative mock surgery was performed strictly following the same drilling protocol and steps of the actual surgery. This step was very critical and beneficial for evaluating the surgical guide’s adaptation and function and for predicting any expected limitations or complications during the actual surgery [17].

In this research, we applied a two-step drilling approach by performing the crestal maxillary osteotomy first followed by the zygomatic bone osteotomy. By allowing long zygomatic drills to pass passively along the crestal part of the osteotomy, we endorsed this approach for two main benefits. First, this helped to minimize the stresses encountered along the long drilling path. Second, it decreased the impediment produced by the limited mouth opening or opposing dentition while using a computer-guided template.

The results of our study revealed no statistically significant differences between the virtual plan and post-operative outcomes, suggesting the use of computer-guided surgical templates in zygomatic implant placement. Furthermore, the deviations detected between the planned and placed implants did not affect the ability of these implants to proceed with the prosthetic phase.

According to this study, it was found that the mean direct linear deviation at the implant platform level was 2.44 ± 1.57 mm (range: 1.31 to 6.21) and at the implant apices 2.32 ± 1 mm (range: 1.01 to 4.16). The mean angular deviation was found to be 3.6˚ ± 1.92 (range: 1.28˚ to 7.06˚). These results fall within the acceptable ranges according to the study conducted by *Van Steenberghe et al.*, which involved placing six fixed-length (45-mm) implants in three formalin-fixed human cadavers. Their results showed linear deviations below 3 mm, and their angular deviations were below 3.5˚ except for only one implant with 6.93˚ [18].

A study conducted by *Vrielinck et al.*, on the other hand, aimed to validate the use of virtual planning software and customized surgical guides for zygomatic and pterygoid implants placement. The mean entry point, exit point, and angular deviations for the zygomatic implants were 2.77 ± 1.61 mm, 4.46 ± 3.16 mm, and 5.14˚ ± 2.59 respectively. All their zygomatic implants followed an intra-sinus trajectory and were placed using surgical guides supported only by the alveolar crest and palatal bones. Notably, this was one of the earliest in vivo studies evaluating the accuracy of guided zygomatic implant placement in 2003 [8].

*Rinaldi et al.*, introducing the novel surgical template design with a lateral cut-off window, reported that deviations from their computerized project to the actual implant positions ranged from 2.5 to 3.5 mm with a mean angular deviation of 4.55˚ [13]. Our study showed comparable or slightly better results, which might emphasize the two major differences between the two studies. First, we relied on the fixation screws to stabilize our guide during drilling as opposed to simple manual seating. Moreover, we had a secondary control point over the long zygomatic drills, which decreased the amount of angular deviation.

Unlike most of the previously mentioned studies, in our results the mean deviation measured at the level of entry points was greater than that measured at the exit points. This might have resulted from the smooth nature of the implant crest module design in addition to the fact that the crestal osteotomy prepared by the final drill (3.6 mm in diameter) was smaller than the platform diameter. In other words, the threaded apical part of the implant had the ability to tap its concentric path while entering the smaller osteotomy. On the other hand, the smoother platform of the implant deviated from its intended position from the more compact palatal bone toward the less resistant buccal direction.

More recent research introducing real-time navigation or static guides fabricated via titanium laser sintering yielded more accurate results. In 2020, *Tao et al.* compared the accuracy of CBCT and MSCT in zygomatic implant dynamic navigation surgery. The comparison of deviations in CBCT and MSCT groups revealed a mean entry deviation of 1.69 ± 0.59 mm vs. 2.04 ± 0.78 mm, apical deviation of 2 ± 0.68 mm vs. 2.55 ± 0.85 mm, and an angular deviation of 2.32˚ ± 1.02 vs. 3.23˚ ± 1.21 [4].

A human cadaveric study, conducted in 2021, assessed the accuracy of zygomatic/pterygoid implant placement using custom-made bone-supported laser-sintered titanium templates. Using the *EZgoma Principle & Guide*, *Grecchi et al.* reported a mean angular deviation of 1.69˚ ± 1.12, a linear deviation of 0.76 ± 0.41 mm at the platform plane, and a linear deviation of 1.35 ± 0.78 mm at the apical plane [12].

In contrast with our research, these two studies’ protocols permitted a fully guided zygomatic implant surgery after the completion of the guided osteotomy, which allowed complete guidance of fixture placement. In our study, the final step of the procedure was carried out in a free-hand manner as implant placement could not be performed through the surgical drill guide due to mechanical limitations.

However, we must compare these more accurate results to our acceptable results with special consideration given to the feasibility and availability of each proposed computer-guided surgical protocol. Unfortunately, laser sintering technology is not yet commonly available on all markets, and when available, templates produced using this technology are more expensive than templates fabricated using acrylic resin [2]. While dynamic navigation could be available, it requires higher facility investment and advanced setup, which would add up further cost to the treatment [8]. The overall cost was an important factor because one of our first goals was to address a less expensive and more affordable treatment alternative.

Another factor affecting our guided surgery accuracy might be the asymmetric distribution of the screws or uneven tightening of the screws. This drawback, in combination with the plastic nature of the guide’s resin material, might have resulted in imbalance of the drilling template creating a possibility of additional deviation [8].

One of the major limitations of this study and probably of all surgically guided studies is the inability to directly compare the guided surgery results in relevance to the free-hand surgery regarding the accuracy [12]. Without directly comparing both methods, it would be difficult to confirm that guided surgery could be considered “more” accurate, predictable, and/or uncomplicated. In zygomatic implant surgery performed free-hand, the surgeon certainly could be inspired by the preliminary virtual planning and assessments, but his/her decisions related to the entry point, path, and exit point of the implants would be made during surgery [13]. Direct choices made during the surgery may represent the best option or treatment modification established for critical situations such as the rehabilitation of severely atrophied maxillary arches.

## Conclusion

Given the major limitation of not being able to place the implants completely guided in their osteotomies, this study can conclude that the use of the double sleeve drill guide allowed favorable control over the tip of the long surgical drill during the zygomatic implant osteotomy preventing injury to adjacent vital structures. Additionally, the use of computer-guided surgical templates allowed the favorable placement of the crestal osteotomy in an acceptable prosthetic position. Overall, the use of computer-guided surgical templates augmented by the double sleeve drill guide may have helped refining zygomatic implants positioning. However, further research and improvement to this protocol should be considered to facilitate a more predictable and accurate surgical outcome along with the prevention of potential complications. To achieve evidence-based results, more studies with larger populations and in the form of randomized clinical comparative trials among different types of computer-assisted zygomatic implant surgeries need to be conducted.

## Data Availability

The datasets used and/or analysed during the current study are available from the corresponding author on reasonable request.
